# The alternative transcription and expression characterization of *Dmc1* in autotetraploid *Carassius auratus*

**DOI:** 10.3389/fgene.2023.1135006

**Published:** 2023-03-28

**Authors:** Xidan Xu, Chongqing Wang, Qingwen Xiao, Xu Huang, Yue Zhou, Xiang Luo, Yuxin Zhang, Xiaowei Xu, Qinbo Qin, Shaojun Liu

**Affiliations:** State Key Laboratory of Developmental Biology of Freshwater Fish, Engineering Research Center of Polyploid Fish Reproduction and Breeding of the State Education Ministry, College of Life Sciences, Hunan Normal University, Changsha, China

**Keywords:** autopolyploidization, meiosis, DMC1, genetic/epigenetic diversity, alternative transcription

## Abstract

Established autotetraploids often have a highly stable meiosis with high fertility compared with neo-autotetraploids. The autotetraploid *Carassius auratus* (4n = 200, RRRR) (4nRR), which stemmed from whole-genome duplication of *Carassius auratus* red var. (2n = 100, RR) (RCC), produces diploid gametes with an adopted diploid-like chromosome pairing in meiosis and maintains the formation of autotetraploid lineages. In this study, we focused on *Dmc1*, a meiosis-specific recombinase during the prophase of meiosis I, and elaborated on the genetic variation, alternative transcription, expression characterization, and epigenetic modification of *Dmc1* in RCC and 4nRR. Two original *Dmc1* from RCC were identified in 4nRR, and two duplicated *Dmc1* differences in genetic composition were observed in 4nRR. Furthermore, we only noticed that one original and one duplicated *Dmc1* were expressed in RCC and 4nRR, respectively. However, both possessed identical gene expression profiles, differential expression of sexual dimorphism, and hypomethylation levels. These results indicated that the specific expression of duplicated *Dmc1* may be involve in the progression of meiosis of the diploid-like chromosome pairing in autotetraploid *Carassius auratus*. Herein, the findings significantly increase knowledge of meiosis of autopolyploid fish and provide meaningful insights into genetic breeding in polyploidy fish.

## 1 Introduction

Polyploid organisms have a profound significance on speciation diversification and biological complexity in the adaptation and evolutionary (Song et al., 2012; Peer et al., 2021). The main distinction between autopolyploid and allopolyploid is the origin of chromosome doubling, which of the former possesses chromosome sets derived from a single taxon while the latter retains a combination of chromosome sets derived from different species (Jackson, 1982; Otto, 2007; Parisod et al., 2010). Autopolyploid is thought to represent evolutionary dead ends that are presented with the multivalent pairing since each chromosome has more than one potential partner, compared to allopolyploids undergoing bivalent pairing at meiosis because of the only pair-up of homologous chromosomes (Otto, 2007; Parisod et al., 2010; Soltis et al., 2010). In polyploids, multivalent that persist to metaphase I is linked to the barrier to the meiotic process and reduced fertility (Lloyd and Bomblies, 2016; Svačina et al., 2020). Actually, established autotetraploids often have a highly stable meiosis with high fertility and do not necessarily form multivalents compared with neo-autotetraploids having low fertility and high levels of aneuploidy (Yant et al., 2013). Autotetraploid *Carassius auratus* (4n = 200, RRRR, 4nRR) derived from the distant hybridization of *Carassius auratus* red var. (RCC) (2n = 100, RR, ♀) × *Megalobrama amblycephala* (BSB) (2n = 48, BB, ♂) that shows diploid-like chromosome pairing in meiosis, which can generate diploid gametes and maintain the autotetraploid lineages (F_1_-F_16_) (Liu et al., 2007; Qin et al., 2014). Moreover, 4nRR possesses four sets of chromosomes derived from RCC. Although there are morphological differences between 4nRR and RCC, they possess standard gonadal structures and arrive at maturation in 1 year (Qin et al., 2018a; Qin et al., 2018b). Consequently, it is crucial to study the molecular basis of adaptation to autotetraploid meiosis.

Meiotic chromosome behavior is essential for fertility across eukaryotes, with basic features that are primarily conserved even across large evolutionary distances (Villeneuve and Hillers, 2001; Mercier and Grelon, 2008; Harrison et al., 2010) and that strictly adhere to genetic regulation processes such as recognition, pairing, synapsis, and recombination of homologous chromosomes, which are prerequisites for balanced segregation of bivalents during meiosis I (Turner et al., 2008; Anderson, et al., 2009; Chowdhury et al., 2009; Pilar and Tomás, 2020). Efficient meiotic recombination establishes the physical linkages or chiasmata between homologous chromosomes which ensure their balanced segregation in the production of gametes (Olivier et al., 2022). DNA meiotic recombinase 1 (*Dmc1*) and *Rad51* assemble on ssDNA at sites of breaks and promote DNA double strand breaks repair by searching for and invading an intact homologous DNA template molecule to involve in meiotic recombination (Xu et al., 2021). In all cases, *Dmc1* deficiency is associated with abnormal homolog pairing and synapsis (Bishop et al., 1992; Couteau et al., 1999). *Dmc1* mutants have severely reduced fertility in Arabidopsis (Yoshida et al., 1998) and resulted in infertility in mice (Pittman et al., 1998). Additionally, high expression of *Dmc1* can contribute to the restoration of bivalent pairing during meiosis in autotriploid *Carassius auratus* (Qin et al., 2019). Based on the available results and given the reproductive property of autotetraploid, what is the effect of autotetraploidization on *Dmc1* in autotetraploid *Carassius auratus*?.

Recent studies have uncovered rapid genomic and genetic changes and epigenetic alterations in autotetraploid *Carassius auratus* (Huang et al., 2020; Wang et al., 2020). Nevertheless, the molecular mechanism of reproductive property that diploid gametogenesis and maintenance of fertility have been noticed is rarely indicated but necessary. Herein, in comparison of genetic variation, expression signature, and epigenetic modification of *Dmc1* between RCC and 4nRR, we obtained a basic understanding of the genetic effects of *Dmc1* in autopolyploidization. Herein, the findings of this study contribute significantly to our understanding of autotetraploid fish meiosis and provide essential insights into genetic breeding in polyploidy fish.

## 2 Methods and materials

### 2.1 Ethics statement

The Institute of Experimental Animals, Hunan Province, China approved all sample protocols in this study. All fish were deeply anesthetized with 100 mg/L MS-222 (Sigma-Aldrich, St Louis, MO, United States) before tissue collection.

### 2.2 Experiment samples

All RCC and 4nRR were selected from the State Key Laboratory of Developmental Biology of Freshwater Fish, Hunan Normal University, China. The generation of RCC and 4nRR was executed during the reproductive season of March 2021 within the Engineering Research Center of Polyploid Fish Breeding and Reproduction of the State Education Ministry, China, located at Hunan Normal University. All individuals were cultured in an open pool (0.067 ha) with suitable pH (7.0–8.5), water temperature (22°C–24°C), dissolved oxygen content (70%), and adequate forage. The cDNA and DNA of Gonad tissue was respectively used to clone CDS sequence and DNA composition. To determine *Dmc1* expression characteristics at different age stages, gonads from RCC and 4nRR fish aged 3, 4, 5, 6, 7, and 11 months were collected, frozen in liquid nitrogen, and stored at −80°C for RNA extraction.

### 2.3 Fluorescence *in situ* hybridization

Chromosome preparations were prepared using kidney tissue of all samples in accordance with Qin et al. (2019a). The probes for fluorescence *in situ* hybridization (FISH) for the specific centromere repeats (263-bp, sequence number JQ086761) of RCC was performed for 4nRR and autodiploid gynogenetic offspring (2n = 100, RR) (G_1_) and amplified *via* PCR using the primers 5′-TTC​GAA​AAG​AGA​GAA​TAA​TCT​A-3′ and 5′-AAC​TCG​TCT​AAA​CCC​GAA​CTA-3′. Detailed FISH steps were performed according to the method described by He et al. (2012).

### 2.4 Specific sequence and expression detection

Total RNA isolation was extracted using Trizol (Thermo Scientific, United States), while the quality and concentration were ascertained by agarose gel electrophoresis and a Nanodrop2000 spectrophotometer (Thermo Scientific, United States), respectively. The cDNA synthesis was achieved using SMARTerTM PCR cDNA Synthesis Kit, and the quality of cDNA was verified by the housekeeping gene (*β-actin*) and agarose gel electrophoresis. The degenerate primers of CDS and DNA full-length amplification of the *Dmc1* gene in RCC were designed based on the nucleotide sequences of Cyprinidae fish and then the CDS and DNA full-length amplification primers of all *Dmc1* in 4nRR were designed based on the genome sequence of 4nRR (unpublished) (Table 1). PCR amplification was respectively configured using cDNA and DNA of gonad as the template, and PCR products were cloned and sequenced to confirm the CDS and DNA specific sequence of *Dmc1* in RCC and 4nRR. The identity and polypeptide sequence alignments of amino-acid sequences of *Dmc1* were utilized with the DNAMAN version7.0. The *qRT*-PCR was carried out as directed by Wang et al. (2021). The *qRT*-PCR was conducted with triplicates. The qPCR-specific primers were designed and distinguished based on the specific SNP in the CDS of each *Dmc1* gene and determined with cloning (Table 1). The results were calculated using the 2^−ΔΔCT^ method (Livak and Schmittgen, 2001). SPSS 20.0 software was used to confirm the one-way Anova test for statistical analysis.

**TABLE 1:** The primer information used for validation of CDS and DNA, methylation primers, and assessment of relative expression levels through *qRT*-PCR.

Primers	Forward sequence (5′–3′)	Reverse sequence (3′–5′)	Purpose to validate
*Dmc1-1*	ATG​AAA​ACT​TTA​GAG​GAC​CAG​G	TTA​GTC​TTC​GGC​ATC​TGT​TAT​T	DNA
*Dmc1-2*	AAC​GTG​GCT​GAA​ATC​AAG​AAA​C	TTA​GTC​TTC​GGC​ATC​TGT​TAT​T	DNA
*Dmc1-3*	ATG​AAA​ACT​TTA​GAG​GAC​CAG​G	TTA​GTC​TTC​GGC​ATC​TGT​TAT​T	DNA
*Dmc1-4*	ATG​AAA​ACT​TTA​GAG​GAC​CAG​G	TTA​GTC​TTC​GGC​ATC​TGT​TAT​T	DNA
*Dmc1*	ATG​AAA​ACT​TTA​GAG​GAC​CAG​G	TTA​GTC​TTC​GGC​ATC​TGT​TAT​T	CDS
*Dmc1*	TCC​ACA​TCA​CAA​CAG​GCA​GTC​TGG​A	CCA​GGA​AGC​TGA​GCG​GTT​ACA​C	*qRT*-PCR
*β-actin*	GCC​CTG​CCC​CAT​GCC​ATC​CT	AGT​GCC​CAT​CTC​CTG​CTC​GA	*qRT*-PCR
*Dmc1-1*	AGA​TGA​TTG​TAG​ATT​TAG​GAG​TT	AATAACCTCATTTTCCRACATA	Methylation
*Dmc1-3*	GGT​GTG​GAG​AGT​ATG​GTT​ATT​ATC	TCC​ATT​TTT​ACT​CTT​ACC​ACA​CAA	Methylation
*Dmc1-4*	GTG​TTT​AGT​TGT​GTT​TTG​TTG​TAT	TCA​TCA​ACT​ACT​ATT​ATT​TTT​CTC​A	Methylation

### 2.5 Methyl-specific PCR analysis

Due to the specific expression and alternative transcription of the *Dmc1* in both RCC and 4nRR gonads, the DNA methylation level in both gonads was detected and analyzed. Comparison and analyses were made based on the expression level at different age stages, genomic DNA extraction of ovary from 5-month-old and testis from 6-month-old in RCC and 4nRR was obtained using TaKaRa MiniBEST universal Genomic DNA Extraction Kit Version. 5.0 (TaKaRa, Japan), while the quality and concentration checking were the same as the RNA. The extracted DNA was sodium bisulfite-modified using the EZ DNA Methylation-Gold™ Kit (Zymo Research, United States). Pseudogenization of *Dmc1-2* sequences and failure of expression were observed in both RCC and 4nRR. Based on the cloned intron sequence of *Dmc1-1* (located in intron 11 and intron 12) in RCC and *Dmc1-1* (located in intron 11 and intron 12), *Dmc1-3* (located in intron 5), *Dmc1-4* (located in intron 3) in 4nRR, the design of methylation primers listed in Table 1, and the prediction of the CpG-rich islands were displayed on the MethPrimer website (http://www.urogene.org/cgi-bin/methprimer/methprimer.cgi). PCR amplification was configured using bisulfite-treated DNA as the template, and PCR products were cloned and sequenced to analyze the methylation status of different *Dmc1*.

### 2.6 Gonadal structure observation

We monitored gonadal development to understand *Dmc1* expression at different age stages in response to the gonadal development in RCC and 4nRR. According to expression characteristics of *Dmc1* in gonad structure at different age stages, ten females (from the age of 3, 5, 7, and 11 months) and ten males (from the age of 3, 4, 6, and 11 months) in RCC and 4nRR were randomly selected for histological observation of gonad structure. Then, detailed steps for observation of gonadal structure were performed following the method described by Qin et al. (2019b).

## 3 Results

### 3.1 Fluorescence *in situ* hybridization

**FIGURE 1:**
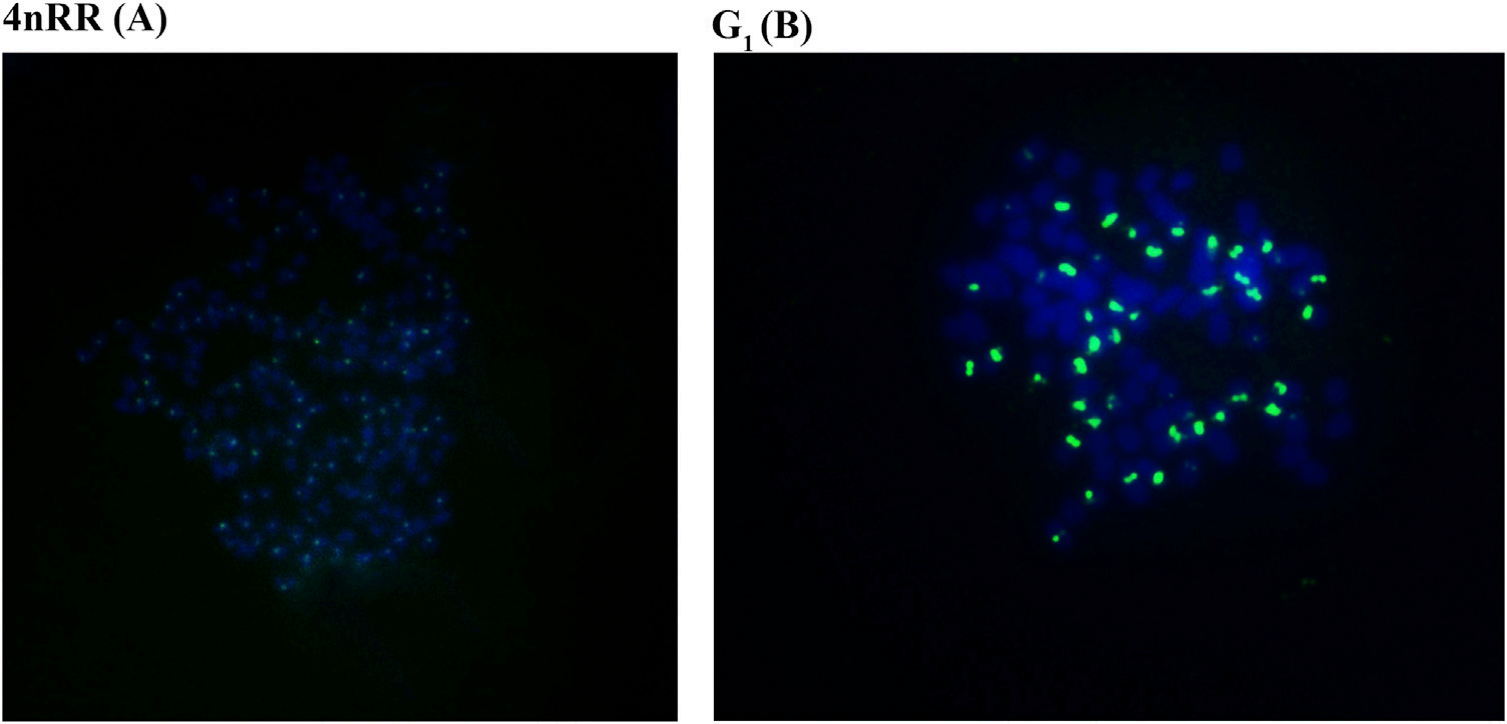
Examination of hybridizing signals in the metaphase chromosomes of in 4nRR and G_1_ by FISH. The arrows indicate the hybridizing signal loci. One hundred specific centromere repeats loci in the chromosomal metaphases of 4nRR **(A)**, and fifty specific centromere repeats loci in the chromosomal metaphases of G_1_ **(B)**. The bars in **(A, B)** denote 3 μm.

Autodiploid gynogenetic offspring were produced by artificial gynogenesis from the eggs of the 4nRR that were activated with UV-treated sterilized sperm of BSB without chromosome doubling treatment. The 263 probe was hybridized to the metaphase chromosomes of 4nRR and G_1_, and the results of FISH were shown in Figure 1. Hybridization of the probe yielded one hundred specific centromere repeats loci in the chromosomal metaphases of 4nRR. And fifty specific centromere repeats loci were detected in the chromosomal metaphases of G_1_. The G_1_ possessed half sets of 4nRR-derived chromosomes, indicating that the chromosome from the eggs of the 4nRR was halved obviously, which is an important sign of meiosis of 4nRR.

### 3.2 The alternative transcription of *Dmc1* in 4nRR

**FIGURE 2:**
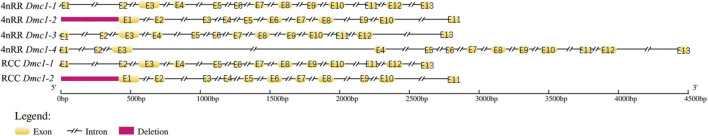
DNA structure character of the *Dmc1* genes in RCC and 4nRR.

**FIGURE 3:**
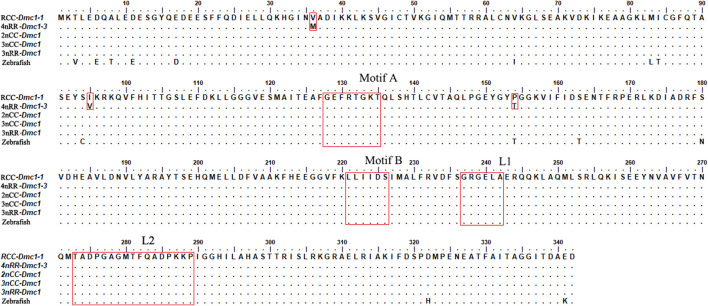
Aligned amino acid (aa) residues of the *Dmc1* genes in RCC, 4nRR, 2nCC, 3nCC, 3nRR, and zebrafish.

Two *Dmc1* copies, including *Dmc1-1* and *Dmc1-2*, were obtained by validation at the DNA level, of which *Dmc1-2* was pseudogenicized because of the deletion of first exon and second exon in both RCC and 4nRR. Moreover, two different duplicated genes (*Dmc1-3* and *Dmc1-4*) possessing complete gene structure were observed in 4nRR (Figure 2). In RCC, *Dmc1-1* and *Dmc1-2* were located on Scaffold271 and Scaffold218 respectively, and *Dmc1-1*, *Dmc1-2*, *Dmc1-3*, *Dmc1-4* were orderly located on chromosome 33A, 42A, 33B, and 42B in 4nRR respectively. However, in the analysis of *Dmc1* transcript, we only noticed *Dmc1-1* (GeneBank: MK140667.1) in RCC and *Dmc1-3* (GeneBank: MH973697.1) in 4nRR with the same length of 1029bp CDS by cDNA cloning, whereas no *Dmc1-1* and *Dmc1-4* transcript were detected in 4nRR. Polypeptide sequence alignments revealed that the identity of *Dmc1-1* in RCC and *Dmc1-3* in 4nRR was 99.1%, while we analyzed and found only three missense-mutation sites (RCC^*Dmc1-1*^/4nRR^*Dmc1-3*^, V^36^/M^36^, I^95^/V^95^, P^154^/T^154^) among them. However, two specific nucleotide binding motifs (A and B) and two DNA binding domains (L1 and L2) were identified in both genes (Figure 3). Furthermore, the genetic and polypeptide sequence of *Dmc1* in diploid *Carassius auratus* (2n = 100, abbreviated as 2nCC), autotriploid *Carassius auratus* (3n = 150, abbreviated as 3nCC), artificial autotriploid *Carassius auratus* (3n = 150, abbreviated as 3nRR) was identical to *Dmc1-1* transcript (Figure 3). The DNA composition and transcript detection demonstrated that the gene variation and alternative transcription of *Dmc1* did emerge following autotetraploidization.

### 3.3 Differential expression of *Dmc1* in gonads

**FIGURE 4:**
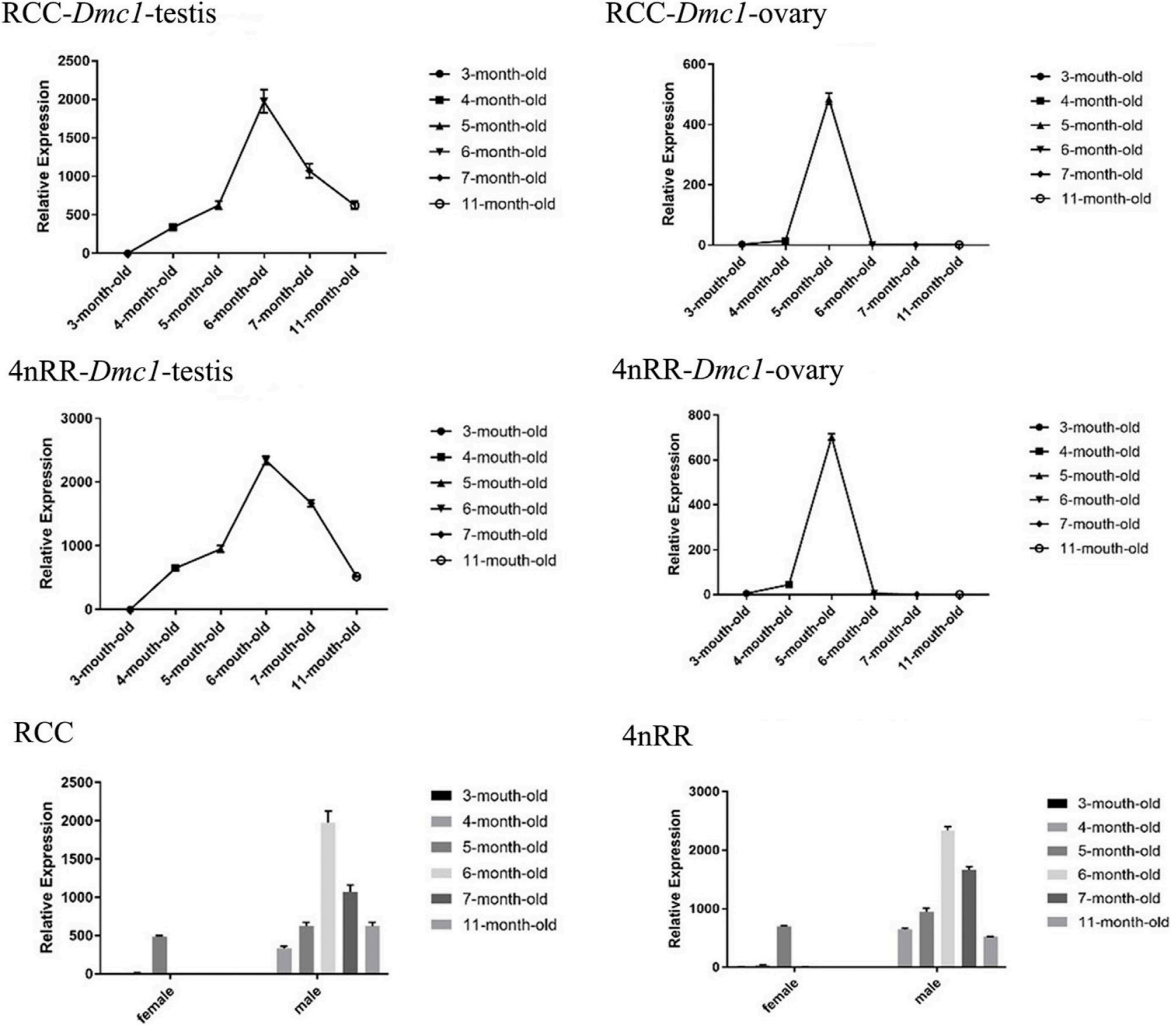
Relative expression levels of *Dmc1* of RCC and 4nRR at different age stages (3, 4, 5, 6, 7, and 11-month-old) of females and males, and the differential expression characteristics between females and males in RCC and 4nRR.

For females in RCC and 4nRR, the ovarian development of individuals differentiated to stage II at 3 months, stage II at 5 months, stage III at 7 months, and stage Ⅳ at 11 months. Moreover, for males in RCC and 4nRR, the testis development of individuals differentiated to stage II at 3 months, stage III at 4 months, stage Ⅳ at 6 months, and stage Ⅴ at 11 months. The identical expression profiles were observed in the *Dmc1-1* of RCC and *Dmc1-3* of 4nRR by quantitative real-time PCR. Figure 4 shows the expression levels of *Dmc1* in gonads of RCC and 4nRR at different age stages. For all female individuals, the *Dmc1-1* in RCC and *Dmc1-3* in 4nRR exhibited identical gene expression profiles in the ovary at different age stages, characterizing by that both genes were expressed only within developmental time point specific. First, the expression level was not detected at 3 months, then the expression level of both genes jumped from extremely low level at 4 months to the highest level at 5 months (*p* < 0.01) and finally dropped acutely to the zero (at 6 months) with no detectable expression level at 7 months and 11 months. However, in the male RCC and 4nRR individuals, the expression level of *Dmc1* can be consistently detected that the expression level gradually increased to the maximum (at 6 months) and then continuously decreased to higher expression level (*p* < 0.01). In addition, in both RCC and 4nRR, different expression level of *Dmc1* within the same development period in female and male can be observed that expression level in testis was significantly higher than the ovary.

**FIGURE 5:**
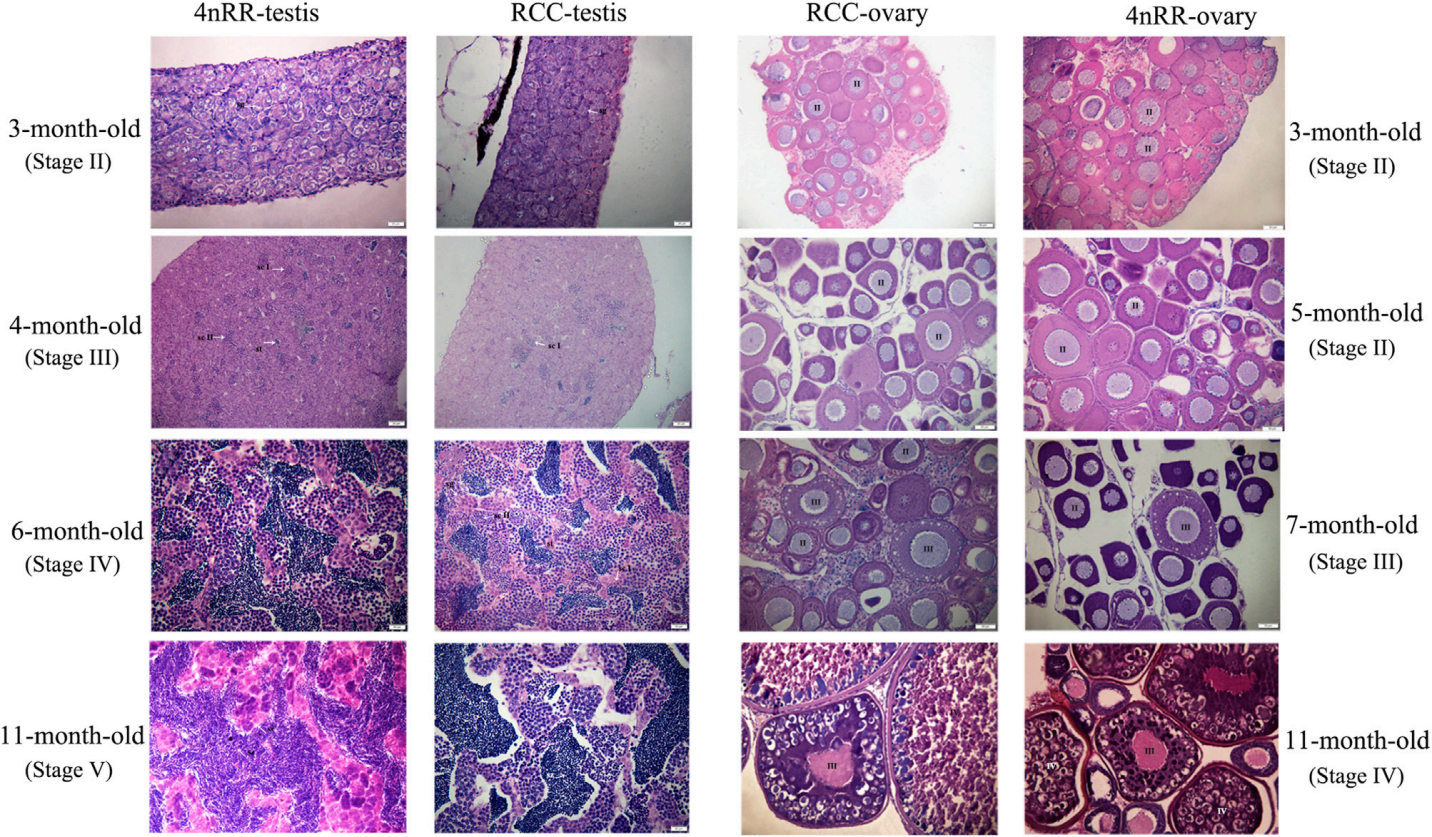
Gonad microstructure of different age stages of females (3, 5, 7, and 11-month-old) and males (3, 4, 6, and 11-month-old) in both RCC and 4nRR. Sg, Spermatogonium; Sc, Sertoli cell; St: Spermatid; Sz: Spermatozoa; ScI: Primaryspermatocytes; ScII: Second spermatocytes; II, phaseⅡoocyte; Ⅲ, phase Ⅲ oocytes; Ⅳ, phase Ⅴ oocytes.

The similar characteristics of gonadal development could be observed in both RCC and 4nRR. Figure 5 shows the gonad microstructure of different age stages of female and male in both RCC and 4nRR. The correlation analysis between expression level of *Dmc1* and ovarian development depicted that large numbers of phase Ⅱ oocytes could be observed when the highest expression was detected in the ovary, however, the abrupt decrease in its expression level and non-expression did not affect the subsequent development of the ovary that the phase Ⅲ oocytes (at 7 months) and phase Ⅳ oocytes (at 11 months) can still be found in the ovary. For males in RCC and 4nRR, spermatogonium at 3 months and a small number of primary spermatocytes, secondary spermatocytes and spermatids at 4 months were formed in the testis as the expression level of *Dmc1* increased. Then large groups of primary spermatocytes, secondary spermatocytes, and some spermatids were noticed at 6 months when the highest expression level was shown. After that, the spermatids and sperms in the testis increased at 11 months when the expression decreased. The above results demonstrated that the differential expression characteristic of *Dmc1* between females and males in 4nRR remained the same as the RCC and *Dmc1* is mainly expressed during the meiosis I.

### 3.4 The alteration of DNA methylation status in *Dmc1*

**FIGURE 6:**
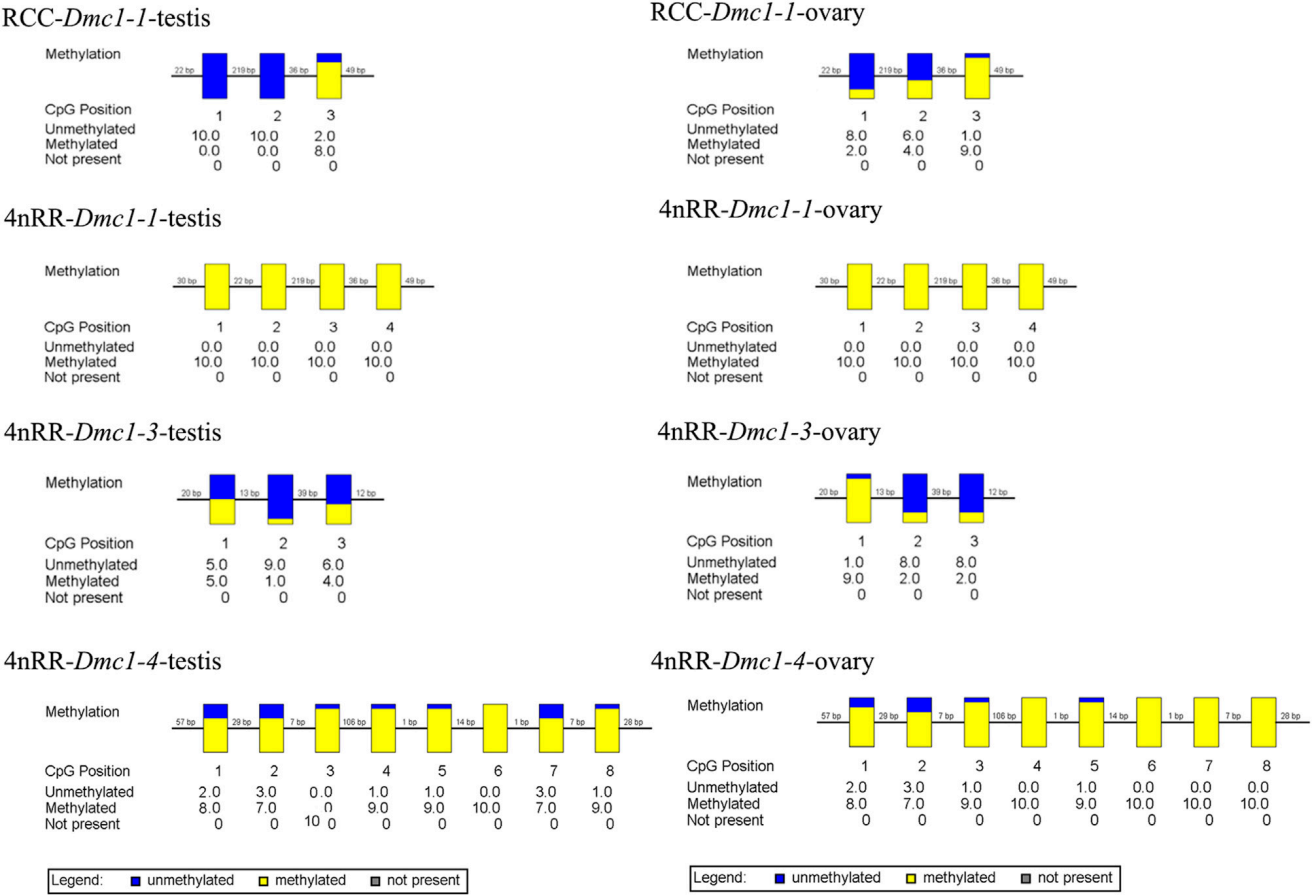
The CpG methylation status of different *Dmc1* in testes (5-month-old) and ovaries (6-month-old) in RCC and 4nRR; Each box displays one CpG position, where the yellow region represents methylation, and the blue region represents no methylation.

Given that, in 4nRR, except for the original *Dmc1-1* and duplicated *Dmc1-4*, the duplicated *Dmc1-3* transcript was only detected, we hypothesized that this case was induced by epigenetic modification alteration, so that we assessed the DNA methylation status of the *Dmc1-1*, *Dmc1-3*, and *Dmc1-4* in 4nRR and *Dmc1-1* in RCC in gonads when the highest expression occurred. Table 2 and Figure 6 depict the methylation status of these genes in the ovary and testis. The results showed that, in RCC, the methylation levels of *Dmc1-1* in 5-month-old ovaries were 0.500 and in 6-month-old testes were 0.267, but they turned into 1.000 in ovaries and testes of 4nRR. In terms of duplicated genes, methylation levels of *Dmc1-3* in ovaries and testes were 0.375 and 0.300, respectively, which were much lower than that of *Dmc1-4*, reaching 0.912 in ovaries and 0.938 testes, respectively. This result suggested that the lowest methylation levels of duplicated *Dmc1-3* was detected, supporting the hypothesis that the alteration of DNA methylation status may affect the transcription and expression of these genes.

**TABLE 2:** Methylation degree of *Dmc1* intron of RCC and 4nRR

Name (type)	Methylation degree
RCC-*Dmc1-1*-Ovary	0.500
RCC-*Dmc1-1*-Testis	0.267
4nRR-*Dmc1-1*-Ovary	1.000
4nRR-*Dmc1-1*-Testis	1.000
4nRR-*Dmc1-3*-Ovary	0.375
4nRR-*Dmc1-3*-Testis	0.300
4nRR-*Dmc1-4*-Ovary	0.912
4nRR-*Dmc1-4*-Testis	0.938

## 4 Discussion

After undergoing genome duplication, polyploids have been considered to alter genetic variation, including recombination, mutation, and chromosomal variation (Song et al., 2012; Peer et al., 2017). The duplicated genes formed by polyploidization might undergo pseudogenization, neofunctionalization, or subfunctionalization (Sandve et al., 2018). Rapid genomic and genetic variations, chromosomal rearrangement, and rDNA sequence changes arose in autopolyploid genome (Qin et al., 2019a; Qin et al., 2019b; Huang et al., 2020). Our results indicated that given the fish-specific whole-genome duplication (FSGD) process, compared with *Dmc1-1*, *Dmc1-2* has been structurally mutated and expression-silenced resulting in its pseudogenization in RCC. The pseudogenization has been considered a special-evolutionary “Relic” in which the retention or elimination contributed to the genome evolution (Seixas et al., 2007; Jia and Liu, 2020). Moreover, in 4nRR, beyond the two original *Dmc1* (*Dmc1-1* and *Dmc1-2*) from RCC, two duplicated *Dmc1* with different DNA lengths and sequences were identified, signifying that the *Dmc1-3* and *Dmc1-4* were induced underlying the autotetraploidization. Newly formed polyploids required reestablishment of genomic balance to overcome genomic incompatibility and transcriptome shock from genomic changes (Hegarty et al., 2006). Therefore, we believed that these flexible variation of *Dmc1* existed in 4nRR may be to overcome threats to survival.

Genome combination and duplication caused massive genetic redundancy (Otto, 2007). Accordingly, proper dose compensation was used to regulate the appropriate amount of gene product to overcome an unstable bottleneck caused by genome duplication (Pala et al., 2008). Dose compensation mechanisms caused changes in genetic and epigenetic modification, resulting in the recombination of genome and expression regulatory networks (Bird et al., 2018). Herein, the transcript detection and expression depicted that the *Dmc1-1* transcript in RCC and *Dmc1-3* transcript in 4nRR were obtained and expressed. However, original *Dmc1-1* and duplicated *Dmc1-4* transcripts were absent in 4nRR, suggesting that the occurrence of alternative transcription of *Dmc1* and specific expression can be induced after autotetraploidization. DNA methylation is a widespread epigenetic phenomenon that is associated with the expression of a gene, and changes in DNA methylation can regulate gene expression (Costello et al., 1994; Ma and Gustafson, 2005; Diez et al., 2014). Additionally, the rapid DNA methylation alteration occurred in some polyploids compared with their diploid progenitors (Fulnecek et al., 2009; Xiao et al., 2013). In this study, the DNA methylation level of *Dmc1-1* in 4nRR was significantly higher than in RCC, correspondingly, the absence of *Dmc1-1* transcript in 4nRR and higher expression level of *Dmc1-1* transcript in RCC can be obtained. In 4nRR, compared with *Dmc1-1* and *Dmc1-4*, *Dmc1-3* exhibited a lowest methylation level and was expressed. We suggested that the alternative transcriptions and specific expressions of *Dmc1* after autopolyploidization can be attributed to the alteration of cytosine methylation, which was also a response to maintain stable expression regulation after genome duplication.

Multivalent pairing is considered to suppresses the diploid gametogenesis during meiosis in polyploids, and yet bivalent pairing is usually facilitated for maintaining the genetic stability of polyploids (Parisod et al., 2010; Shahid et al., 2013). Consequently, the establishment of polyploidy and lineages are tightly associated with diploid-like chromosome pairing behavior (Comai et al., 2000; Soltis et al., 2009). The appearance of bivalent pairing in meiosis depends on the precise pairing of homologous chromosomes. Homologous recombination and synaptonemal complexes are the main factors affecting homologous chromosome pairing (Kleckner, 1996). *Dmc1* is specifically expressed during meiosis I and is mandatory for regulating the homologous chromosome pairing and synapsis, which of the lower expression or mutation can result in synapsis disorders and cause dysplasia in gonadal development and gametogenesis disorder (Bishop, 1994; Chen et al., 2016). The lower expression of *Dmc1* in the testis was associated with male sterility of cattle-yak (Xian et al., 2010). Herein, *Dmc1-3* in 4nRR and *Dmc1-1* in RCC exhibited identical expression profiles in the testis and ovary, respectively. Furthermore, both genes were specifically expressed in phase Ⅱ oocytes and sustainedly expressed in spermatocytes. Phase II oocytes is the protoplasm growth period of primary oocytes that is the growth period of cytoplasm and cell nucleus after the oocyte stops mitosis, and Phase Ⅲ oocytes and phase Ⅳ oocytes are regarded as the grand period of growth of primary oocytes. Accordingly, we suggested that *Dmc1* and *Dmc3* were specifically expressed during the meiotic I in RCC and 4nRR respectively, while a similar result was found previously in different ploidy cyprinid fishes (Tao et al., 2008). The above features indicated that *Dmc1-3* in 4nRR may have the same function as the *Dmc1-1* in RCC, and it was involved in developing gonads in a ploidy-independent way, which may be crucial in promoting normal meiosis in RCC and 4nRR. However, the differential expression of sexual dimorphism of *Dmc1* in RCC and 4nRR can be observed, characterizing by higher sustainable expression level in the testis from primary spermatocyte to the spermatogenesis stage while the specific expression level in the primary oocyte of meiosis Ⅰ, and expression level in testis was significantly higher than the ovary within the same development period, which can be observed in *Litopenaeus Vannamei* (Okutsu et al., 2010) and *Acipenser dabryanus* (Xiang et al., 2019). Consequently, we speculated that differential expression might be related to the meiotic cycle of spermatogonia and oogonia, and spermatogonium sustainably produces spermatids and sperm since spermatogonia exist in different generations. More research is needed based on this study data to determine whether the differential expression is due to developmental properties or other regulatory functions.

## Data Availability

The original contributions presented in the study are included in the article/supplementary material, further inquiries can be directed to the corresponding author.
